# Fatal Persistent Cavitary Mycobacterium avium Complex Pulmonary Disease in a Non-HIV Patient With Prior Malignancy

**DOI:** 10.7759/cureus.114787

**Published:** 2026-08-19

**Authors:** Lina Ataya, Garrett Beck, Aceil Shamieh

**Affiliations:** 1 Internal Medicine, Henry Ford Genesys Hospital, Grand Blanc, USA; 2 Internal Medicine, Michigan State University College of Human Medicine, Michigan, USA

**Keywords:** bronchiectasis, cavitary lung disease, malignancy, mycobacterium avium complex, nontuberculous mycobacteria, pulmonary mac, recurrent infection, respiratory failure

## Abstract

*Mycobacterium avium* complex (MAC) pulmonary disease most commonly occurs in patients with structural lung abnormalities or impaired cellular immunity and generally follows a chronic, slowly progressive course. Risk factors for disease progression and recurrence include cavitary disease, persistent structural lung abnormalities, and incomplete treatment courses. Although recurrence is common, progression to respiratory failure and death remains uncommon among non-HIV patients. We report the case of a 71-year-old female with a history of recurrent MAC pulmonary infection, bronchiectasis, colon cancer treated with surgical resection and chemotherapy, remote bone malignancy, hypertension, hyperlipidemia, and iron deficiency anemia who presented with progressive dyspnea, productive cough, weight loss, and generalized weakness. Computed tomography of the chest demonstrated extensive bilateral cavitary lesions, pulmonary nodules, and bronchiectasis. Sputum cultures confirmed recurrent MAC infection. Despite initiation of guideline-directed therapy with azithromycin, rifampin, and ethambutol, the patient experienced progressive respiratory deterioration requiring mechanical ventilation and ultimately died from respiratory failure. This case highlights the potential for recurrent MAC pulmonary disease to follow an aggressive and fatal course in non-HIV patients, particularly in the setting of structural lung disease, prior malignancy, and prolonged interruptions in therapy. Early recognition and sustained treatment adherence remain critical to improving outcomes.

## Introduction

*Mycobacterium avium* complex (MAC) is an environmental nontuberculous mycobacterium that commonly causes pulmonary disease in patients with structural lung abnormalities, HIV infection, and hematologic malignancies, as well as in individuals with impaired cell-mediated immunity [1]. In non-HIV populations, MAC pulmonary disease typically follows a chronic and indolent course characterized by gradual radiographic progression and persistent respiratory symptoms [1,2]. Risk factors include cavitary disease, persistent structural lung abnormalities, and incomplete treatment courses [3-5]. Although severe disease may occur, rapid progression to respiratory failure and death despite initiation of guideline-directed therapy remains rare in non-HIV patients with MAC pulmonary disease [2,5].

We present a case of fatal persistent cavitary MAC pulmonary disease in a non-HIV patient with bronchiectasis and prior malignancy whose disease rapidly progressed to respiratory failure despite initiation of guideline-directed therapy, highlighting an unusually aggressive clinical course.

## Case presentation

A 71-year-old Caucasian female with a past medical history significant for childhood bone cancer requiring lower extremity amputation, colon cancer treated with surgical resection and chemotherapy, a history of MAC pulmonary infection, iron deficiency anemia, hypertension, and hyperlipidemia presented with progressively worsening shortness of breath over the preceding five to six months. Her symptoms initially occurred with exertion but progressed to dyspnea at rest. She also reported an unintentional 25-pound weight loss, generalized weakness for the past six months, and a productive cough with yellow sputum.

The patient reported a prior diagnosis of MAC pulmonary disease approximately 10 years earlier following treatment for colon cancer. She denied a history of HIV infection. Treatment for MAC had been intermittent because of medication intolerance and financial barriers. She had previously been prescribed inhaled amikacin but was unable to tolerate therapy. Approximately two years prior to presentation, she discontinued antimycobacterial treatment because it became cost-prohibitive.

**Figure 1:**
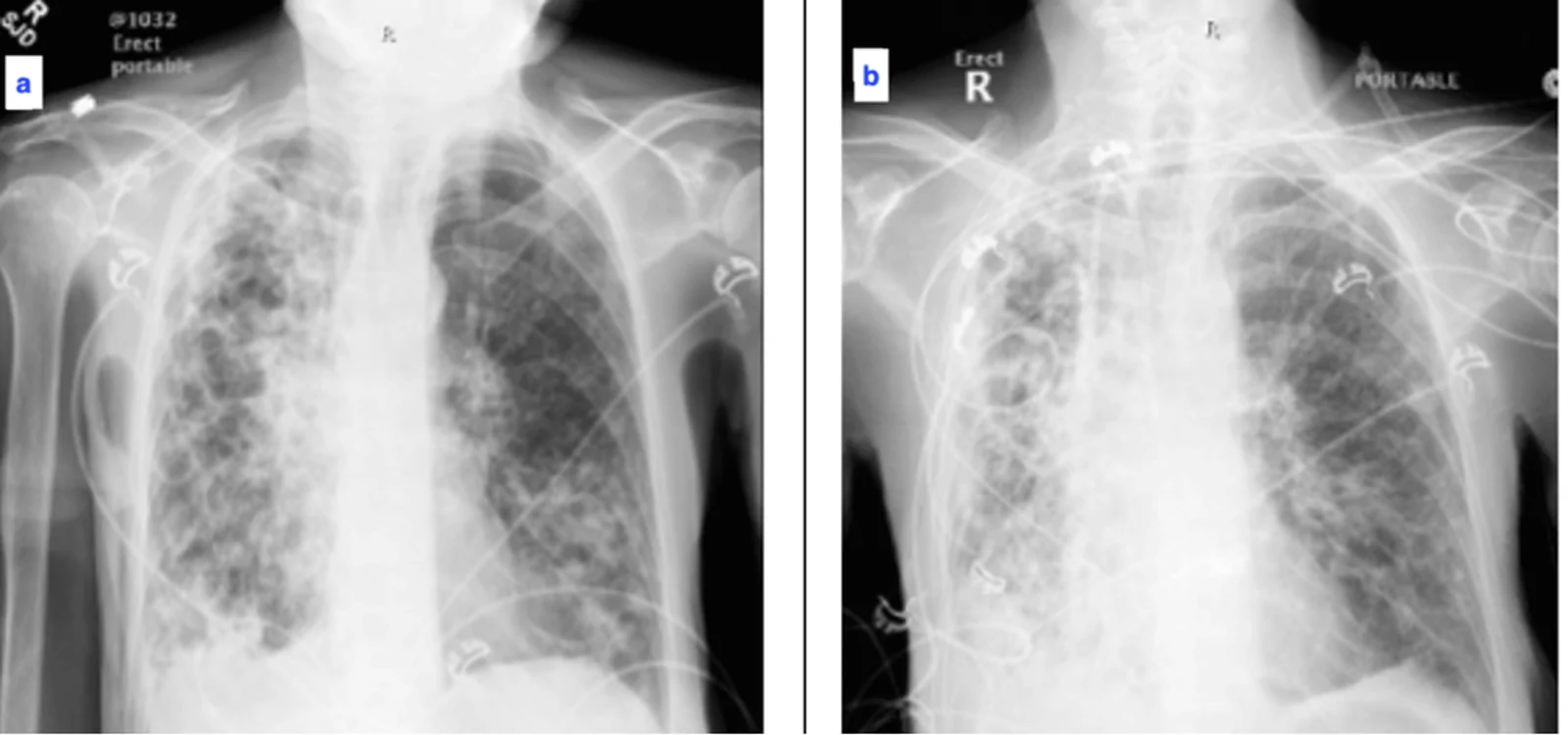
Chest radiographs demonstrating interval progression of pulmonary disease. (a) Initial radiograph demonstrating bilateral pulmonary infiltrates, more prominent in the right lung. (b) Follow-up chest radiograph obtained one week after admission demonstrating worsening aeration and increasing nodular infiltrates involving the left lingula and left lower lobe.

On presentation, laboratory evaluation demonstrated leukocytosis (white blood cell count 18.9 × 10³/µL). Vital signs were notable for a blood pressure of 136/63 mmHg, heart rate of 105 beats per minute, respiratory rate of 30 breaths per minute, and oxygen saturation of 94% while receiving supplemental oxygen at 2 L/min via nasal cannula. Chest radiography demonstrated bilateral pulmonary infiltrates, more prominent in the right lung (Figure 1a). Electrocardiography showed sinus tachycardia with nonspecific T-wave abnormalities. Initial management included intravenous fluid resuscitation and empiric antimicrobial therapy with vancomycin and cefepime for suspected bacterial pneumonia.

**Figure 2:**
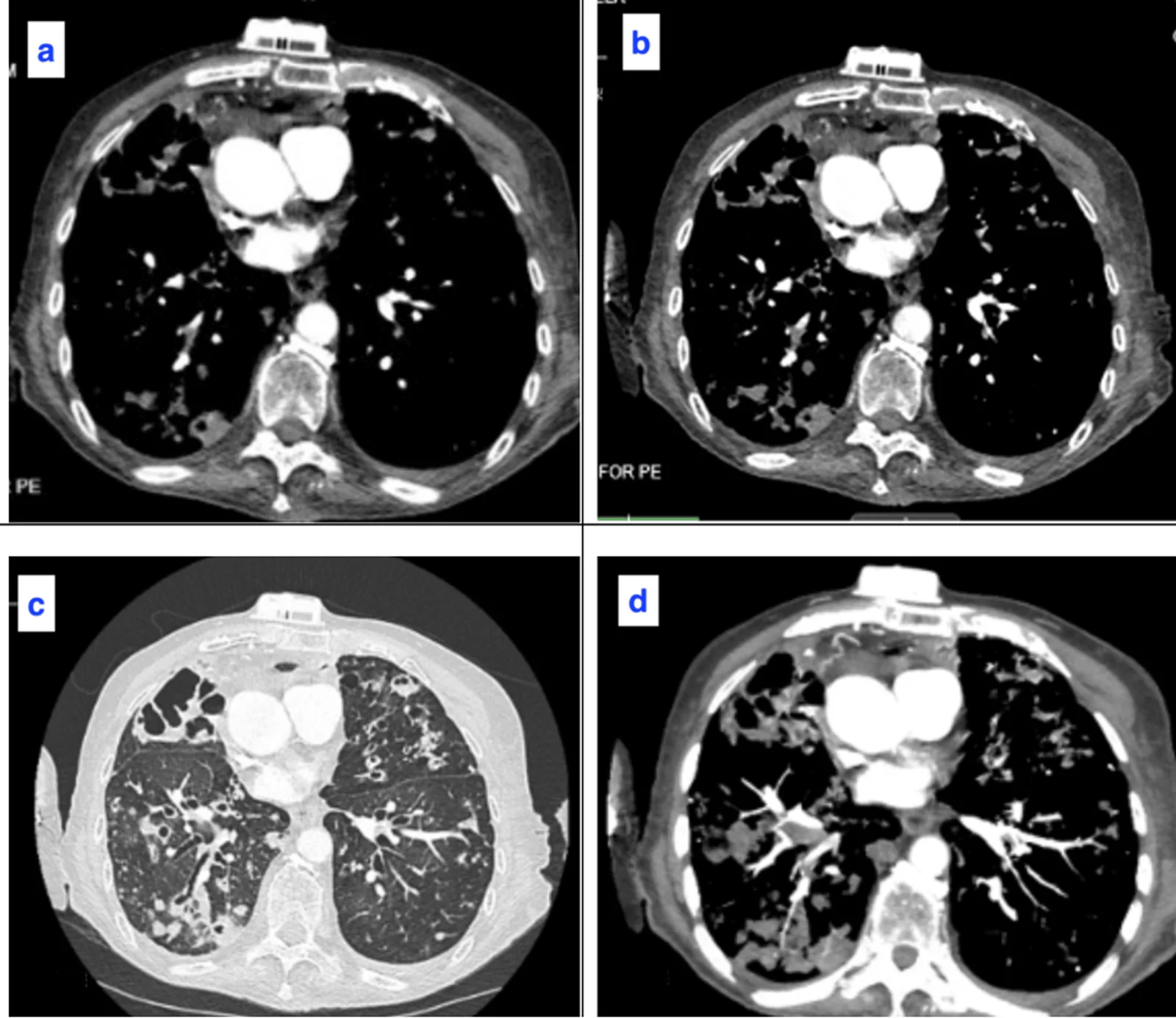
Contrast-enhanced computed tomography (CT) of the chest obtained at admission. (a–d) Axial CT images demonstrating extensive bilateral cavitary pulmonary lesions, most pronounced in the right lung, with surrounding ground-glass opacities. Numerous bilateral pulmonary nodules, areas of consolidation, and bilateral bronchiectasis are also present. No pleural effusion or pneumothorax is identified.

Computed tomography angiography of the chest demonstrated no evidence of pulmonary embolism but revealed extensive bilateral cavitary pulmonary lesions with numerous bilateral pulmonary nodules and masses. Surrounding ground-glass opacities and bilateral bronchiectasis were also present (Figure 2a-2d). Sputum acid-fast bacillus cultures subsequently grew *Mycobacterium avium*, which, in conjunction with the patient’s clinical presentation and radiographic findings, supported the diagnosis of MAC pulmonary disease. Additional infectious workup was negative, including testing for HIV, methicillin-resistant *Staphylococcus aureus* (MRSA), respiratory viral pathogens, *Streptococcus pneumoniae*, and* Legionella* species.

During hospitalization, the patient developed worsening fatigue, fever, persistent tachycardia, and increasing leukocytosis. Cefepime was transitioned to intravenous Zosyn (piperacillin 4 g and tazobactam 0.5 g), and intravenous linezolid 600 mg was added. Following consultation with the infectious disease service, treatment for MAC pulmonary disease was initiated with azithromycin 250 mg, rifampin 600 mg, and ethambutol 15 mg/kg. Intravenous amikacin was considered but was not initiated because of renal failure. An 18-month course of therapy was recommended by the infectious disease service.

Following initiation of MAC-directed therapy, the patient experienced continued clinical deterioration. Repeat chest radiography one week after admission demonstrated worsening aeration and increasing nodular infiltrates involving the left lingula and left lower lobe (Figure 1b). Her hypoxemia progressively worsened, ultimately requiring endotracheal intubation and mechanical ventilation. She subsequently developed septic shock requiring norepinephrine and vasopressin for hemodynamic support. Her ICU course was complicated by severe lactic and metabolic acidosis, acute kidney injury, hyperkalemia, and NSTEMI. Given her worsening clinical status and persistent shock, antimicrobial coverage was broadened to meropenem and vancomycin, while MAC-directed therapy was continued. Supportive management included intravenous fluid resuscitation and bicarbonate infusion for severe acidosis. Despite these interventions, she developed progressive respiratory and circulatory failure and ultimately died during hospitalization.

## Discussion

This case demonstrates an unusually aggressive presentation of persistent* *MAC pulmonary disease in a non-HIV-infected patient with bronchiectasis and prior malignancy.

MAC infection is typically associated with structural lung disease and immunosuppression and generally follows a chronic, slowly progressive course [1,2]. However, our patient developed extensive bilateral cavitary destruction with rapid progression to respiratory failure despite initiation of recommended multidrug therapy.

Structural lung disease likely played a central role in disease persistence and progression. Bronchiectasis is a recognized risk factor for MAC pulmonary disease because impaired mucociliary clearance promotes chronic microbial colonization and creates a favorable environment for persistent nontuberculous mycobacterial infection [1]. In addition, the patient had a prior history of MAC pulmonary disease with subsequent treatment interruption, which may have contributed to disease persistence and progression [3,4].

The presence of extensive bilateral cavitary disease likely contributed substantially to the patient's poor outcome. Cavitary MAC pulmonary disease represents the most severe radiographic phenotype and has been associated with higher organism burden, delayed culture conversion, increased recurrence rates, and greater mortality than nodular bronchiectatic disease [2,5]. The patient's advanced age and widespread cavitary destruction on admission represented important adverse prognostic features and reflected advanced pulmonary disease [5,6].

Treatment interruption was another important contributor. Effective management of MAC pulmonary disease requires prolonged multidrug therapy, typically continued for at least 12 months after culture conversion [1]. Previous studies have reported recurrence rates approaching 40-50% following successful treatment [3,4]. In this case, treatment was repeatedly interrupted because of medication intolerance and financial barriers, including discontinuation of inhaled amikacin and eventual cessation of antimycobacterial therapy. These interruptions may have contributed to disease persistence and progression. Additionally, antimicrobial susceptibility data were unavailable; therefore, macrolide resistance could not be excluded in the setting of her history of intermittent therapy. This case further highlights the significant impact that medication access and treatment affordability may have on long-term outcomes in chronic nontuberculous mycobacterial disease.

The patient's history of colon cancer treated with chemotherapy and remote bone malignancy may have contributed to her overall clinical complexity; however, their independent contribution to MAC disease progression remains uncertain. While MAC infection is most commonly associated with structural lung disease, HIV infection, and hematologic malignancies, emerging evidence suggests that patients with solid-organ malignancies may also have an increased risk of nontuberculous mycobacterial disease [7,8,9]. Previous studies have suggested possible long-term alterations in cellular immunity following malignancy or cancer-directed therapy [7,10]. Although a causal relationship cannot be established in this case, the patient's oncologic history may have contributed to disease progression.

Alternative diagnostic considerations included pulmonary tuberculosis, fungal infection, metastatic malignancy, and necrotizing bacterial pneumonia. However, the identification of* M. avium* on sputum AFB cultures, in conjunction with the clinical and radiographic findings, supported the diagnosis of MAC pulmonary disease.

This case highlights that advanced MAC pulmonary disease can occasionally deviate from its expected indolent course and present with rapidly progressive cavitary destruction, respiratory failure, and death [2,5,6]. Clinicians should maintain a high index of suspicion for disease progression in patients with structural lung disease, prior MAC infection, cavitary lesions, and barriers to treatment adherence.

## Conclusions

Advanced cavitary* *MAC pulmonary disease can rarely progress to fatal respiratory failure in patients without HIV infection. Structural lung disease, extensive cavitary destruction, treatment interruption, and delayed re-engagement in care may contribute to poor outcomes. Early recognition, close longitudinal follow-up, sustained access to and tolerance of guideline-directed multidrug therapy, and efforts to address barriers to medication access are essential to improving outcomes in high-risk patients.
